# A network-based pharmacological investigation to identify the mechanistic regulatory pathway of andrographolide against colorectal cancer

**DOI:** 10.3389/fphar.2022.967262

**Published:** 2022-08-30

**Authors:** Balakarthikeyan Janani, Mayakrishnan Vijayakumar, Kannappan Priya, Jin Hee Kim, Ayman Geddawy, Mohammad Shahid, Mahmoud H. El-Bidawy, Sameer Al-Ghamdi, Mohammed Alsaidan, Mohammad Hassan Abdelzaher, Abubucker Peer Mohideen, Thiyagarajan Ramesh

**Affiliations:** ^1^ Department of Biochemistry, PSG College of Arts and Science (Autonomous), Affiliated to Bharathiar University, Coimbatore, Tamil Nadu, India; ^2^ Department of Integrative Bioscience and Biotechnology, Sejong University, Seoul, South Korea; ^3^ Department of Basic Medical Sciences, College of Medicine, Prince Sattam Bin Abdulaziz University, Al-Kharj, Saudi Arabia; ^4^ Department of Pharmacology, Faculty of Medicine, Minia University, Minia, Egypt; ^5^ Department of Physiology, Faculty of Medicine, Cairo University, Cairo, Egypt; ^6^ Family and Community Medicine Department, College of Medicine, Prince Sattam Bin Abdulaziz University, Al-Kharj, Saudi Arabia; ^7^ Internal Medicine Department, College of Medicine, Prince Sattam Bin Abdulaziz University, Al-Kharj, Saudi Arabia; ^8^ Department of Medical Biochemistry, Faculty of Medicine, Al-Azhar University, Assiut, Egypt

**Keywords:** colorectal cancer, molecular target, network pharmacology, protein-protein interaction, gene ontology

## Abstract

Traditional cancer treatments have posed numerous obstacles, including toxicity, multiple drug resistance, and financial cost. On the contrary, bioactive phytochemicals used in complementary alternative medicine have recently increased attention due to their potential to modulate a wide range of molecular mechanisms with a less toxic effect. Therefore, we investigated the potential regulatory mechanisms of andrographolide to treat colorectal cancer (CRC) using a network pharmacology approach. Target genes of andrographolide were retrieved from public databases (PharmMapper, Swiss target prediction, Targetnet, STITCH, and SuperPred), while targets related to CRC were retrieved from disease databases (Genecards and DisGeNet) and expression datasets (GSE32323 and GSE8671) were retrieved from gene expression omnibus (GEO). Protein-protein interaction networks (PPI) were generated using STRING and Cytoscape, and hub genes were identified by topology analysis and MCODE. Annotation of target proteins was performed using Gene Ontology (GO) database DAVID and signaling pathway enrichment analysis using the Kyoto Encyclopedia and Genome Database (KEGG). Survival and molecular docking analysis for the hub genes revealed three genes (PDGFRA, PTGS2, and MMP9) were involved in the overall survival of CRC patients, and the top three genes with the lowest binding energy include PDGFRA, MET, and MAPK1. MET gene upregulation and PDGFRA and PTGS2 gene downregulation are associated with the survival of CRC patients, as revealed by box plots and correlation analysis. In conclusion, this study has provided the first scientific evidence to support the use of andrographolide to inhibit cellular proliferation, migration, and growth, and induce apoptosis by targeting the hub genes (PDGFRA, PTGS2, MMP9, MAPK1, and MET) involved in CRC migration and invasion.

## Introduction

Colorectal cancer accounts for around 10% of all cancer diagnoses and cancer-related deaths globally each year. It is the second most prevalent cancer in women and the third most frequent cancer in men. Incidence and fatality rates are around 25% lower in women than in men (Dekker et al., 2019). Overall, about 4%–5% of the global population suffers from colorectal cancer. Moreover, certain personal characteristics can act as risk factors for polyps’ formation, as they can increase the likelihood of developing the disease (Mármol et al., 2017). The likelihood of developing CRC is influenced by many modifiable factors, including obesity, smoking, and eating red meat. Reduced colorectal cancer risks have been associated with physical activity, postmenopausal hormone therapy, nonsteroidal anti-inflammatory drugs, and vegetable and fruit consumption (Johnson et al., 2013). Initiation, promotion, and progression are the stages in the formation of CRC. Epithelial cells in the intestinal mucosa become irreversibly damaged by genetic damage, resulting in subsequent neoplastic transformation (Sawicki et al., 2021). A cancerous growth (cancer) is generated during the promotion phase. In the progression stage, benign carcinoma cells turn malignant and develop aggressive characteristics as well as the capacity to metastasize (Fares et al., 2020). The options for treatment of primary and metastatic cancers (mtCRC) have greatly expanded recently. Among these options are laparoscopic surgery for early-stage CRC, aggressive resection of metastatic CRC (such as lung and liver metastases), radiotherapy for RC, and neoadjuvant and palliative chemotherapy (Dekker et al., 2019; Afshar et al., 2020). The traditional therapies for CRC include surgery and chemotherapy (Woods and Turchi, 2013). Chemotherapy for CRC involves the use of some known drugs with limited clinical applications because of their toxic side effects and resistance to prominent anti-cancer drugs. New treatments for CRC that are less hazardous are therefore required as an alternative or in addition to current methods (Moradi et al., 2019). Chemotherapeutics can cause cancer cell death by generating DNA damage or activating numerous signaling pathways, such as cell cycle arrest, global translation inhibition, and DNA repair (Woods and Turchi, 2013). The impacts of cytotoxicity, drug resistance, and severe effects are the primary issues linked with chemotherapy (Huang et al., 2019). According to the World Health Organization (WHO), around 80% of the world’s population relies on conventional therapies (Aiello et al., 2019).

Phytochemicals, naturally occurring substances in plants, are an important resource for developing new therapeutics and being used as cancer therapies as well. Phytochemicals significantly impact molecular pathways involved in cancer development and progression. Numerous specific strategies are utilized. These include enhancing antioxidant levels, inactivating carcinogens, inhibiting proliferation, causing cell cycle arrest and death, and suppressing immune activity (Choudhari et al., 2019). Due to its numerous medical uses, *Andrographis paniculata*, also known as Kalmegh in India, is largely recognized as a beneficial plant. It is commonly known as the “king of bitters” and is widely distributed throughout India and Sri Lanka. It is a member of the Acanthaceae family (Paul et al., 2019). For centuries, the aerial parts, roots, and entire plant of *A. paniculata* have been used as traditional medicine in Asia to treat a variety of ailments. Traditional healthcare professionals have used it to treat inflammation, pyrexia, stomachaches, and intermittent fevers (Okhuarobo et al., 2014). An important bioactive phytoconstituent in *A. paniculate* is andrographolide, found primarily in the leaves (Jayakumar et al., 2013). It exhibits a variety of biological properties, including immunomodulatory, antiviral, and anti-inflammatory properties, and is often used to treat sore throats, fevers, diarrhea, and inflammatory illnesses such as colitis (Shimura et al., 2021). Additionally, andrographolide has been shown to limit cancer cell motility and invasion by inhibiting the production of proteins or cellular signaling pathways involved in cancer metastasis.

In drug development, the strategy of generating multi-target therapies against complex diseases such as diabetes and cancer is growing rapidly. In this regard, network pharmacology describes disease processes as networks that are best addressed by a combination of medications that function together. A network is a visual representation of a dataset that highlights the connections between the nodes. These nodes, which stand in for genes, proteins, small molecules, and other entities that can interact in the system being represented, are connected by edges, which stand in for the characteristics of the interactions, to form a graph (Berger and Iyengar, 2009). To gain a better understanding of how herbal medicines operate, network pharmacology is increasingly being used (Noor et al., 2022). Network pharmacology aims to identify genes associated with compounds and diseases, create a network of protein-protein interactions (PPIs), and then evaluate and display the network (Gao et al., 2021; Niu et al., 2021). Through the mechanistic exploration of effects on multiple disease pathways, network pharmacology represents an effective method for selecting and elucidating the synergistic effects of bioactive chemicals (Obaidullah et al., 2022). The hub genes are the highly interconnected nodes in the PPI network that are very likely to play a role in crucial biological regulation. Important biological processes are revealed using hub gene and Protein-Protein Interaction (PPI) network analysis, which offers effective methods for identifying important molecular pathways in cancer biology. Hub genes obtained from network pharmacology either represent the molecular targets of active compound (Lv and Li, 2019) or prognostic genes (Bian et al., 2019). In this study, the molecular regulatory mechanism of action of andrographolide against colorectal cancer was predicted with the aid of network pharmacology and docking studies.

## Materials and methods

### Target collection for andrographolide

The potential targets of andrographolide were explored and assessed utilizing public databases such as PharmMapper (http://www.lilab-ecust.cn/pharmmapper/index.html), Swiss target prediction (https://www.swisstargetprediction.ch/), Targetnet (http://targetnet.scbdd.com/home/index/), STITCH (http://stitch.embl.de/) and SuperPred (https://prediction.charite.de/subpages/target_prediction.php). In all these databases species was limited to *Homo Sapiens* once the target has been predicted. The gene list of the targets obtained were standardized using UniProtKB (https://www.uniprot.gov/).

### Identification of critical genes in CRC from disease database

Proto-oncogenes and tumour suppressor genes make up the two main classes of genes known as “cancer-critical genes,” which are responsible for the development of cancer (Alberts et al., 2002). The critical genes related to Colorectal cancer was obtained using the keywords “colorectal cancer,” “colorectal adenomas,” and “colorectal neoplasms” in five publicly available databases such as DisGeNET (http://www.disgenet.org/) using the cutoff “score_gda >0.1,” GeneCards (https://www.genecards.org/) only the genes with the relevance score greater than 30. The target gene information connected to CRC was collected and incorporated for the species “*H. sapiens*.” The integrated gene list was then uploaded in UniProtKB to obtain gene name and ID, then the duplicates were removed.

### Identification of critical genes in CRC from expression dataset

GSE32323 and GSE8617 were the two-microarray expression dataset selected for identifying differentially expressed genes in CRC. The datasets were retrieved from NCBI GEO database (http://www.ncbi.nlm.nih.gov/geo/). GSE32323 consists of expression profile of 17 colorectal cancer tissue samples and 17 normal tissue samples (34 samples in total), GSE8671 consists of 32 pair (64 samples in total) of samples corresponding to Colorectal adenoma and normal tissues. GEO2R, an online tool incorporating R/Bioconductor and the Limma package v3.26.8 was used to examine the raw gene expression data. To determine the DEGs among patients with CRC and controls, we used the built-in methods in GEO2R, such as the *t*-test and Benjamini and Hochberg (false discovery rate) and force normalization of the datasets were applied to eliminate redundancy and minimize modification errors of data. Volcano plot was plotted for CRC VS Normal to represent significant up and down regulated genes. The genes with adjusted *p* value < 0.01 and a log_2_FC ≥ 2 were filtered and considered as DEGs. The DEGs obtained from the above process was used for further investigation. The heat map of the two datasets were plotted using SRplot (https://www.bioinformatics.com.cn/) and the network of the top 100 DEGs obtained from two datasets were plotted using Cytoscape. The Targets obtained from disease target database and GEO datasets (Top 100 genes) were integrated and a Venn diagram was created to identify the genes shared by targets of andrographolide and CRC targets.

### PPI network construction

Using STRING a web-based tool (http://string-db.org) (v11.5), we established a PPI network to examine the relationships between DEGs acquired from the datasets (Manoochehri et al., 2021). The minimum interaction score was set at 0.09. The protein nodes that do not have any interaction with other nodes were removed from the network. Insights into biological mechanisms can be derived from the analysis of functional interactions between proteins. To identify the hub genes and key modules the PPI network from STRING was imported to Cytoscape (version 3.5.1; http://www.cytoscape.org). Considering the networks were scale-free, the biological importance of genes associated with degree centrality was examined to better understand their functionality. Network parameters such as degree, closeness and betweenness was analyzed using analyze network option in Cytoscape. Degree describes the link between one node with their adjacent nodes, the degree cut off (degree > 10) was set to find the hub genes in the network. From the resulting PPI network, we used the MCODE plugin from Cytoscape to detect intersected clusters using parameters Node cutoff score-0.2, K-score-2 and Max Depth-100.

### Pathway and functional enrichment analysis

Gene Ontology (GO) analysis is now a widely used method of analyzing genomic data, especially large-scale transcriptomic data. To determine the biological function of target proteins and cancer-related pathways we performed Gene Ontology (GO) and Kyoto Encyclopedia of Genes and Genomes (KEGG) enrichment analysis using The Database for Annotation, Visualization, and Integrated Discovery (DAVID, https://david.ncifcrf.gov/, ver. 6.8) (Manoochehri et al., 2021). The enriched GO terms and pathways with a False Discovery Rate (FDR) of less than 0.01 was selected for further visualization. Bubble graph and of top 20 significant GO terms (BP, CC, and MF) and KEGG pathways were plotted using SRplot (https://www.bioinformatics.com.cn/).

### Molecular docking

To predict the interaction between hub genes and andrographolide, molecular docking was performed (Bahmani et al., 2021). PDB structures of the 19 hub genes (PDB IDs of genes in Table 1) were retrieved from RCSB Protein Data Bank (http://www.rcsb.org), whereas the 2D structure of andrographolide was retrieved from PubChem database (https://pubchem.ncbi.nlm.nih.gov/), the SDF format was converted into PDB format using Open Babel. The water molecules and native ligand groups from the protein structure were removed using Discovery Studio Visualizer, which then predicted the active site for most efficient ligand binding. Further processing of proteins and docking of proteins (10 docking runs were performed for each protein) were performed using AutoDock Tools. A visualization of the 2D and 3D interactions between the top three protein-andrographolide complexes with the lowest binding energy was created using the Discovery Studio Visualizer.

**TABLE 1:** Degree, betweenness, closeness, and binding energy values of 19 hub genes.

Gene ID	Probe ID	Name	Degree	Betweenness	Closeness	Binding energy (kcal/mol)
EGFR	5FED	Epidermal growth factor	18	0.013505	1	−6.97
MTOR	4JT6	Mammalian target of rapamycin	18	0.013505	1	−6.97
SRC	3ELD	Proto-oncogene tyrosine-protein kinase	18	0.013505	1	−7.74
HRAS	6ZL3	HRAS proto-oncogene, GTPase	18	0.013505	1	−7.14
IL6	1N26	Interleukin-6	17	0.011354	0.947368	−6.07
ESR1	7MSA	Estrogen receptor1	17	0.010683	0.947368	−8.72
JAK2	6VGL	Janus kinase 2	17	0.012629	0.947368	−8.28
AKT1	3MVH	AKT serine/threonine kinase 1	16	0.011358	0.9	−8.06
KDR	6GQQ	Kinase insert domain receptor	16	0.005546	0.9	−5.99
MAPK1	7PQV	Mitogen-activated protein kinase 1	15	0.004576	0.857143	−7.85
KIT	6GQJ	KIT proto-oncogene, receptor tyrosine kinase	15	0.004987	0.857143	−7.97
CASP3	7SEO	Caspase3	15	0.00394	0.857143	−7.30
PTPN11	3B7O	Protein tyrosine phosphatase non-receptor type 11	15	0.007317	0.857143	−6.32
MMP9	4H1Q	Matrix metallopeptidase 9	15	0.00394	0.857143	−8.85
MET	6UBW	MET proto-oncogene, receptor tyrosine kinase	15	0.00467	0.857143	−6.33
PTGS2	6BL4	Prostaglandin-endoperoxide synthase 2	14	0.00297	0.818182	−8.86
PIK3R1	5ITD	Phosphoinositide-3-kinase regulatory subunit 1	14	0.00685	0.818182	−7.42
TLR4		Toll-like receptor 4	13	0.004612	0.782609	
PDGFRA	6JOL	platelet-derived growth factor receptor alpha	10	8.75E-04	0.692308	−9.01

### Survival analysis

In GEPIA, a log-rank test is used to analyze overall survival by considering gene expression. In order to analyze the relationship between the hub genes and overall survival of patients with CRC, Kaplan-Meier (K-M) survival curves were plotted using GEPIA2 (http://gepia2.cancer-pku.cn/#index) for COAD (Colon Adenocarcinoma) and READ (Rectum Adenocarcinoma) database. *p* values of <0.05 were considered statistically significant. The top three genes with lowest binding energy from molecular docking and genes involved in survival of CRC patients were integrated and used for further analysis.

### Correlation and expression analysis

The correlation analysis between the six genes (three genes from survival analysis and three from docking) was performed using GEPIA after log2 transformation. In terms of significance levels, values less than 0.05 considered (significant-*), 0.01 (highly significant-**), and 0.001 (very highly significant-***). Based on user-defined sample selections and methods, GEPIA dynamically plots expression profiles of a given gene. A comparison of the expression profiles between tumor samples and adjacent normal samples was performed by GEPIA on the Cancer Genome Atlas (TCGA) database for colon adenocarcinoma (COAD and READ) and rectum adenocarcinoma (READ) with statistical significance *p* < 0.01.

## Results

### Target screening for andrographolide and CRC

**FIGURE 1:**
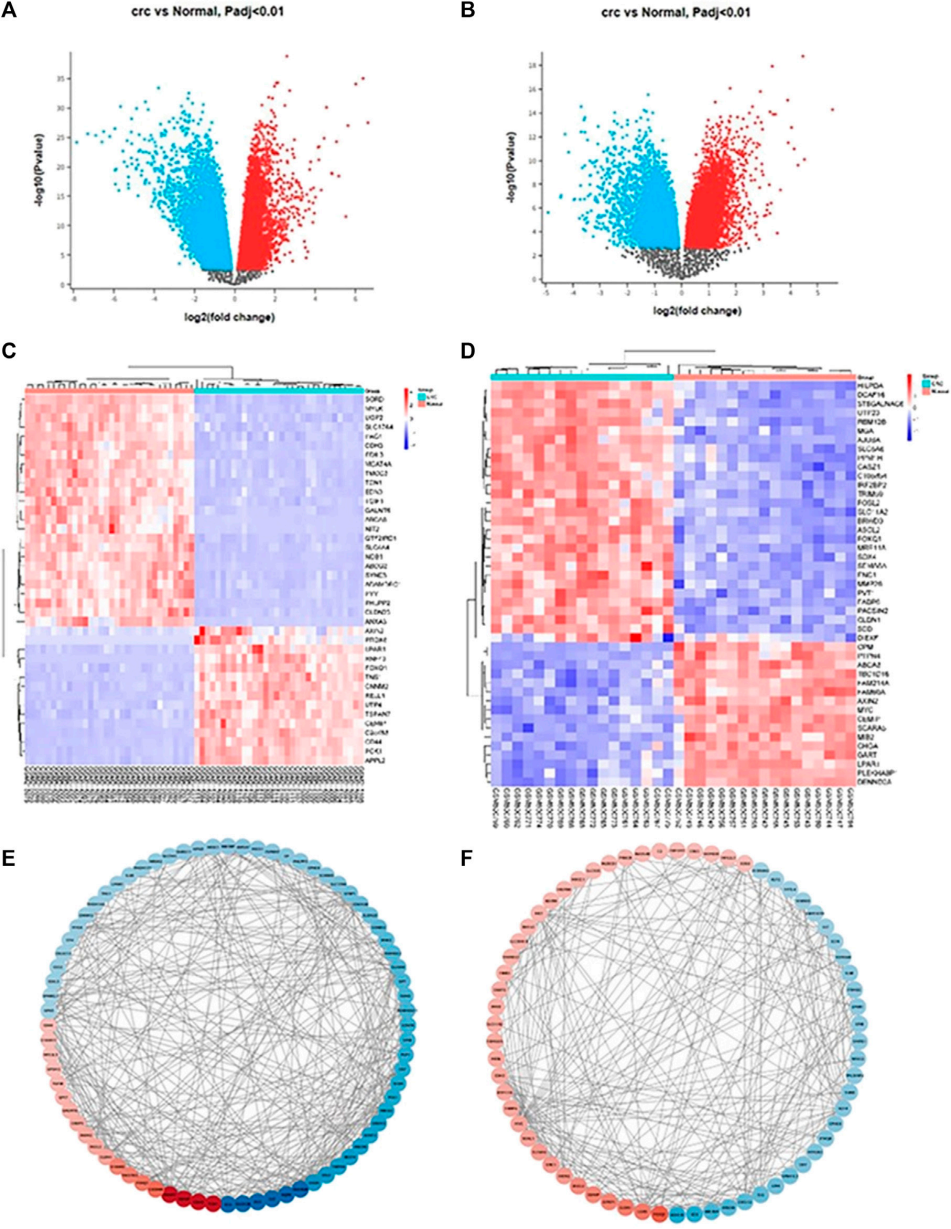
**(A)** GSE32323 volcano plot of differentially upregulated and downregulated genes with significance *p* < 0.01, Upregulated genes are shown in red colour, Downregulated genes are shown in blue colour; **(B)** GSE8671 volcano plot of differentially upregulated and downregulated genes with significance *p* < 0.01, Upregulated genes are shown in red colour, Downregulated genes are shown in blue colour; **(C)** GSE32323 top 100 up and down expressed genes heatmap, Upregulated genes are shown in red colour, Downregulated genes are shown in blue colour. **(D)** GSE8671 top 100 up and down expressed genes heatmap, Upregulated genes are shown in red colour, Downregulated genes are shown in blue colour; **(E,F)** represents network diagram (arranged according to LogFC value) of top 100 DEGs plotted using STRING and Cytoscape.

**FIGURE 2:**
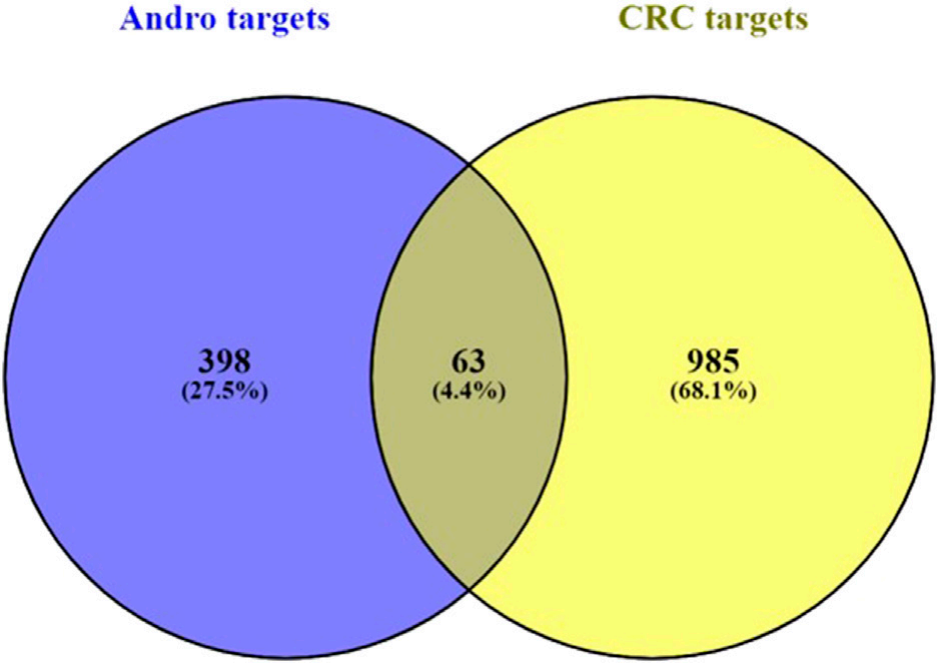
Overlap co-expression Venn map between targets of andrographolide and CRC with a total of 63 genes.

After uploading the appropriate format of andrographolide, we were able to retrieve a total of 398 targets from the target database. From the disease database, 985 CRC targets were obtained with the cut-off levels for Genecards and DisGeNet being GDA > 30 and score GDA > 0.3. Following the application of the cut-off adj *p*-value <0.01 and log2FC ≥ 2 to the datasets GSE32323 and GSE8671, the number of DEGs obtained was 441 and 873, respectively. In GSE32323, 145 upregulated and 306 downregulated genes were observed, and in GSE8671, 197 upregulated and 678 downregulated genes were observed. The volcano plots were created from the DEGs in both datasets (Figure 1A). In (Figure 1B), the heatmaps of both datasets are displayed (Figures 1C, D). The network of top 100 DEGs (Figures 1E, F) from each dataset was plotted using Cytoscape. To proceed with the analysis, the top 100 DEGs from each dataset were selected. 63 genes were obtained after finding the overlap between andrographolide targets and CRC targets (Figure 2).

### PPI network Analysis

**FIGURE 3:**
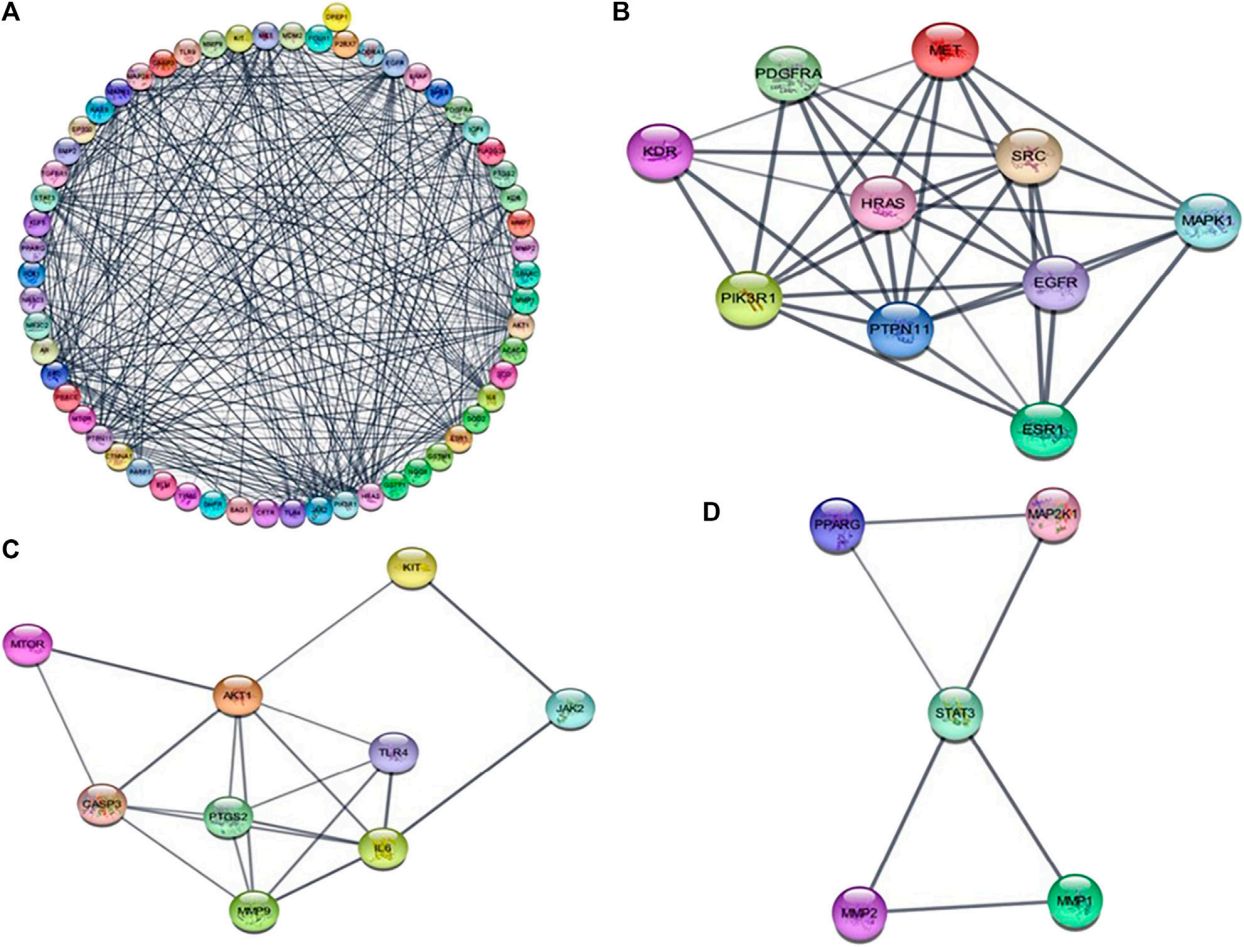
**(A)** protein-protein interaction network diagram of 63 intersected DEGs between andrographolide and CRC. **(B–D)** represents those three modules obtained from the PPI network using MCODE plugin in cytoscape.

**FIGURE 4:**
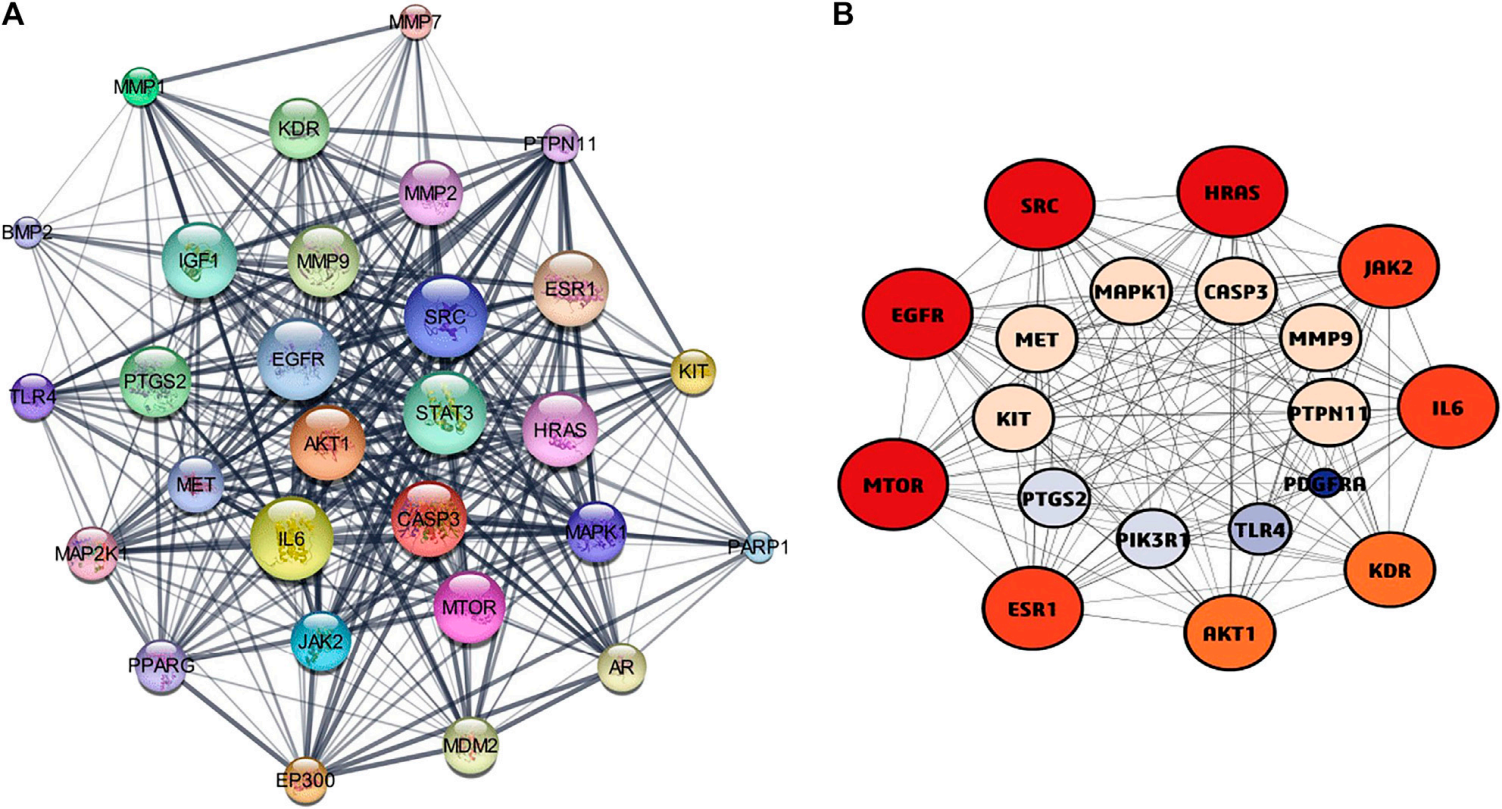
**(A)** protein-protein interaction network diagram of Hub genes. **(B)** The top Hub genes screened by the both colour and degree of nodes (red-highest degree, blue-lowest degree).

The STRING online tool was used for the analysis of protein-protein interaction network of 63 overlapped genes (Figure 3A) with a minimum interaction score of 0.700 (highest confidence). Cytoscape software was used to construct the PPI network, the network consists of 60 nodes and 240 edges. Three modules (Figures 3B–D) were obtained from the network using MCODE application in Cytoscape software, the modules with score ≥4 and genes with degree ≥10 (Table 1) were selected for further analysis. 19 genes were obtained after filtering, these genes were considered as hub genes. A subnetwork for 19 hub genes were created (Figures 4A,B).

### Functional and pathway enrichment analysis (GO and KEGG analysis)

**FIGURE 5:**
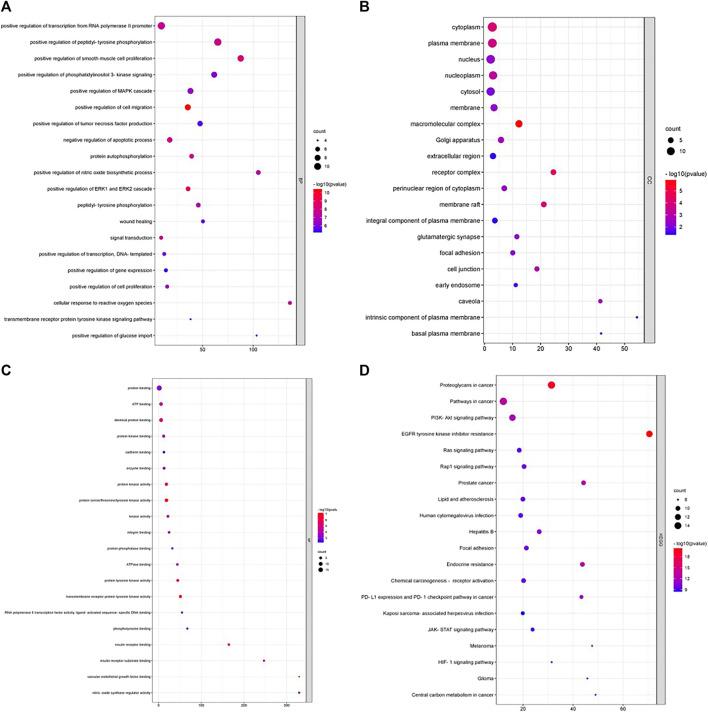
**(A–D)** represents the bubble chart of top 20 enriched DEGs for biological process, cellular compartment, molecular function by GO and KEGG analysis.

To understand the functions and pathways of the hub genes in CRC, GO functions and pathways were analyzed with DAVID. A variety of GO enrichment terms were identified, including 204 Biological Processes (BP), 27 Cellular Components (CC) and 43 Molecular Functions (MF). A bubble chart was created displaying the top 20 enriched terms for each GO function (Figures 5A–C) and KEGG pathway (Figure 5D). The hub genes may regulate cell migration, ERK1 and ERK2 cascade, smooth muscle cell proliferation and, negative regulation of apoptotic process through molecular functions like transmembrane receptor protein tyrosine kinase activity, tyrosine kinase activity and, protein kinase activity in cellular components like macromolecular complex, membrane raft, cytoplasm, and plasma membrane. A total of 133 KEGG pathways has been found to be associated with the hub genes, among 133 pathways EGFR tyrosine kinase inhibitor resistance, Proteoglycans in cancer, Pathways in cancer, Prostate cancer, Endocrine resistance, and PI3K-Akt signaling pathway were the most significantly enriched pathways. 14 hub genes were found to be involved with PI3K-Akt signaling pathway. From the pathway study it is evident that andrographolide may exert its anticancer activity through regulating ERK1 and ERK2 and MAPK cascade *via* PI3K-Akt signaling pathway.

### Molecular docking analysis

**FIGURE 6:**
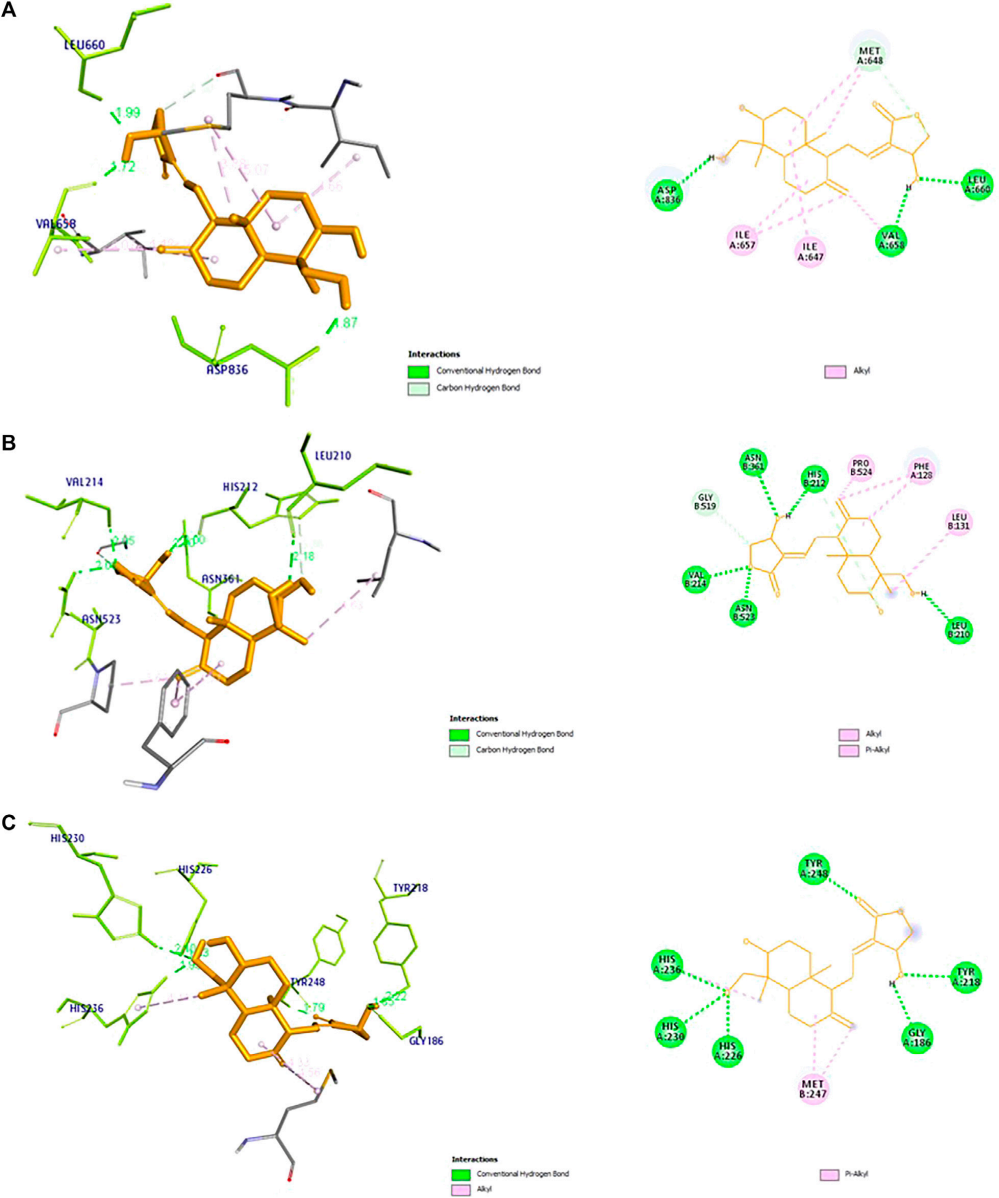
3D and 2D receptor ligand interaction diagram of top three genes with Lowest binding energy for **(A)** PDGFRA, **(B)** PTGS2, and **(C)** MMP9. The diagram represents the information including, type of bond between the receptor and ligand, and their bond distance.

The docking results of the 19 hub genes is shown in (Table 1). Lower binding energy values indicate more stable structures. Among the target genes PTGS2, ESR1, and PDGFRA found to have lowest binding energy. The 2D and 3D interactions between andrographolide and three target genes is shown in (Figure 6).

### Survival, expression, and correlation analysis

**FIGURE 7:**
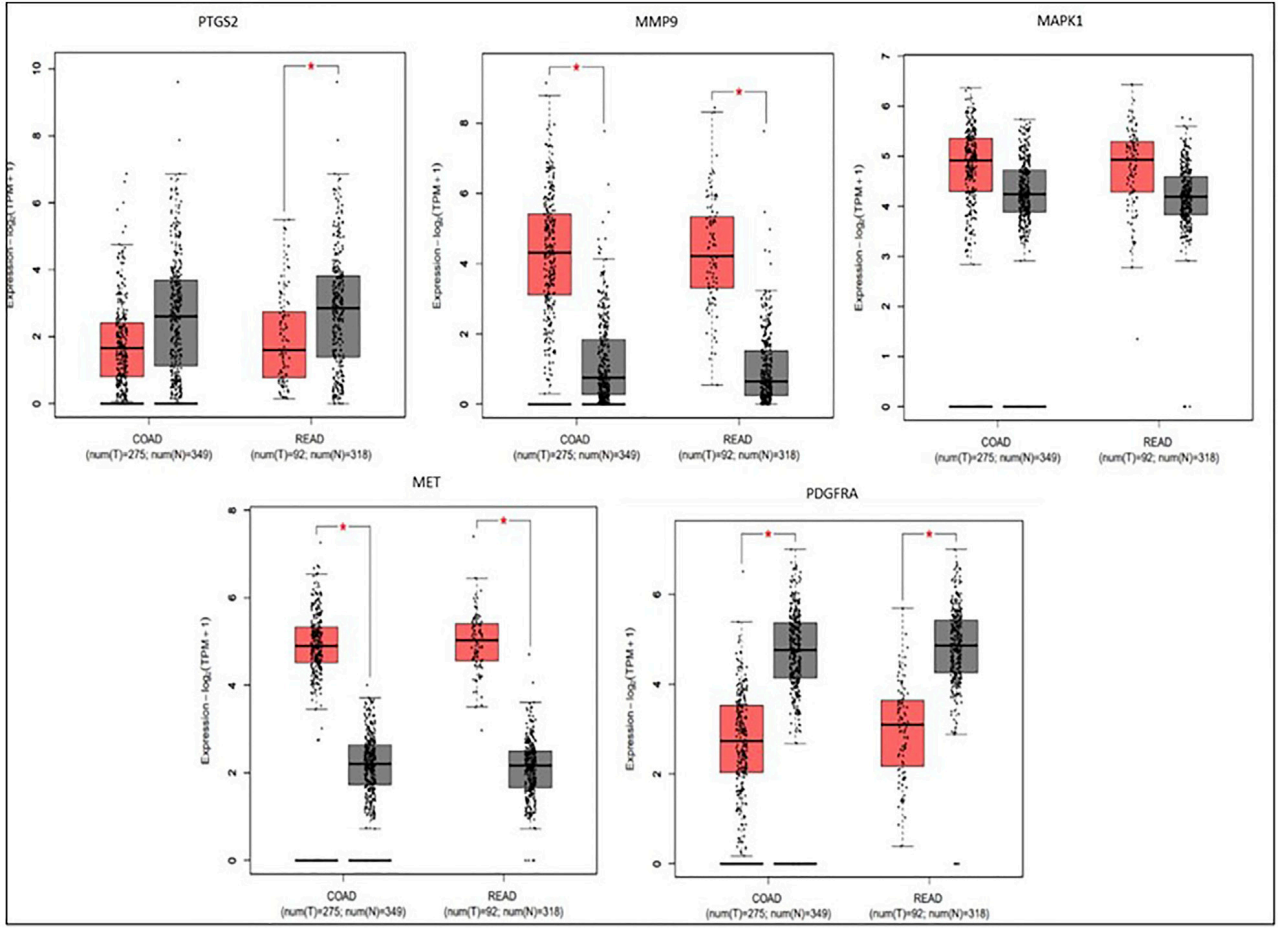
Survival plots for three genes (MET, PTGS2, and MAPK1) which was found to be involved in the overall survival of CRC patients among 19 hub genes.

**FIGURE 8:**
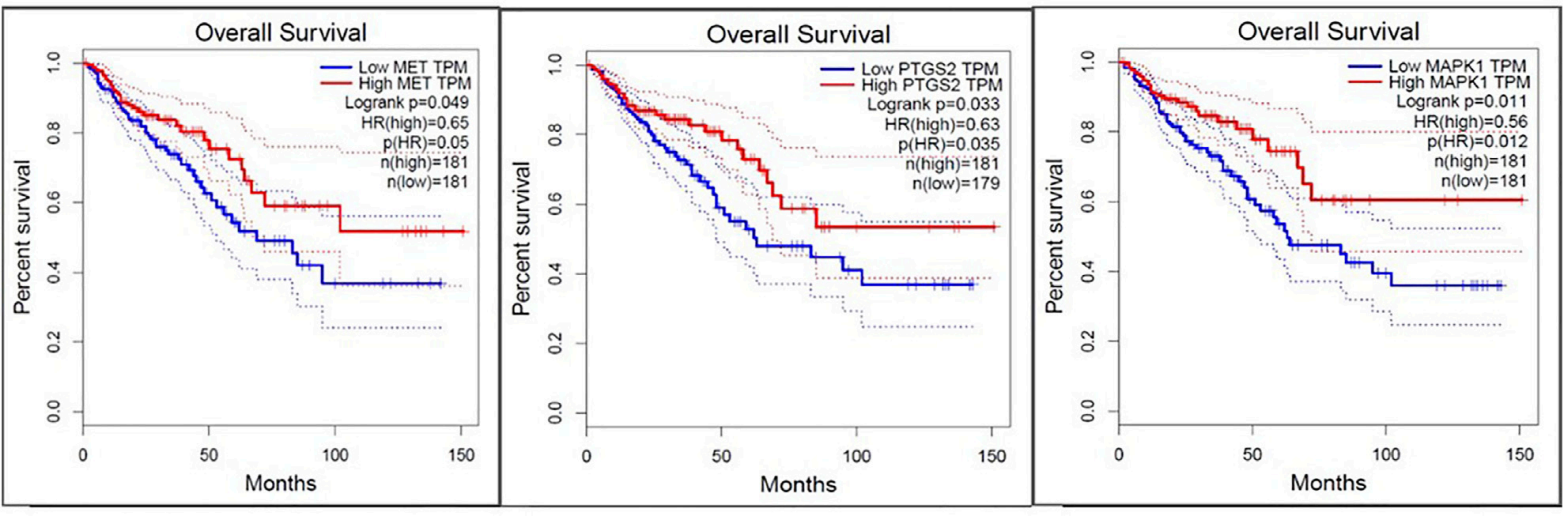
Analysis of the correlations between the five genes PTGS2, MMP9, MET, MAPK1, and PDGFRA using Pearson’s coefficient analysis at *p* < 0.05. Positive correlation between the genes is shown in green colour, negative correlation between the genes is shown in red color, and genes with no correlation is shown in blue colour *-indicates the correlation is significant and ***-indicates the correlation is highly significant.

**FIGURE 9:**
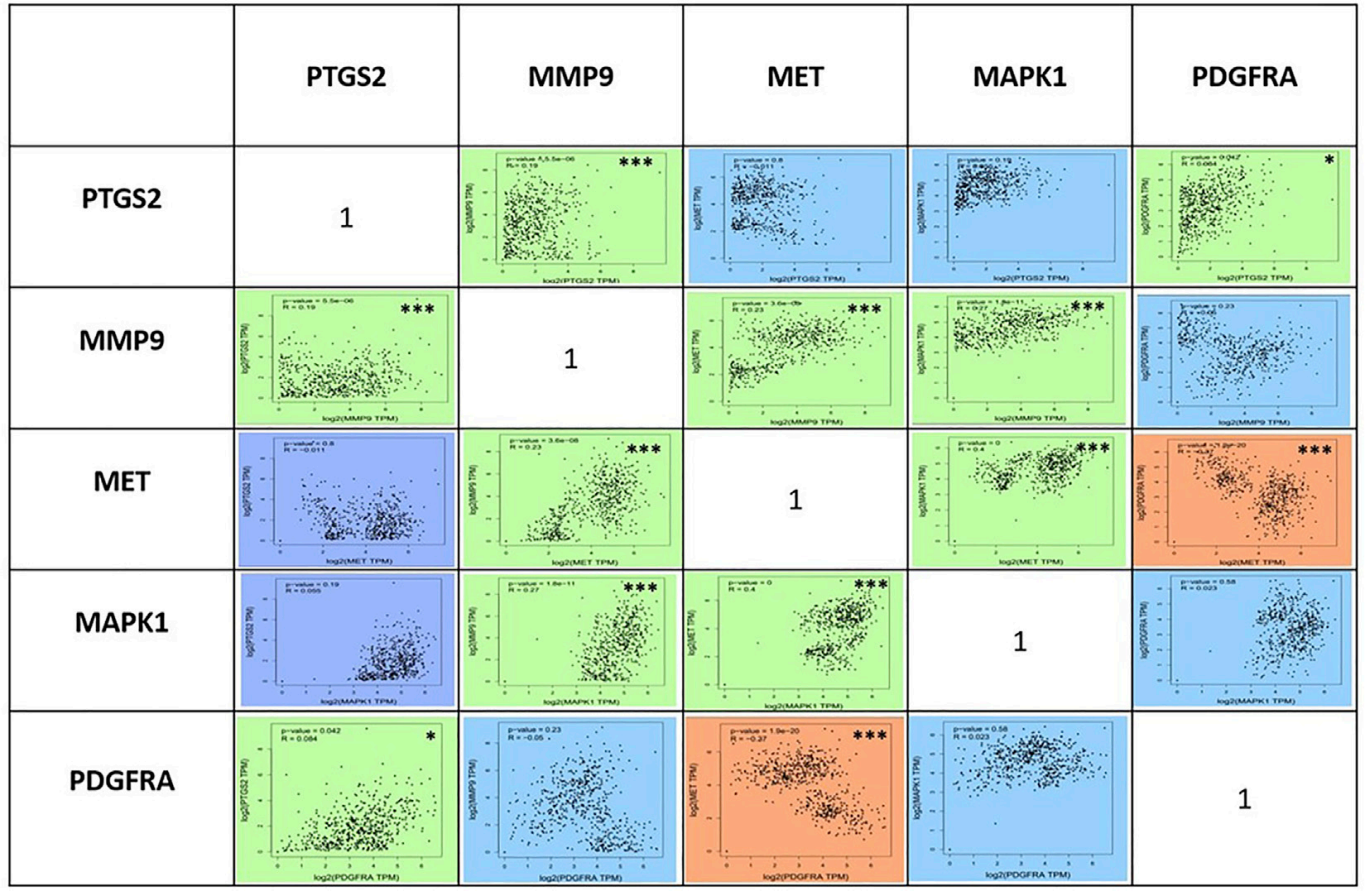
Box plots depicting expression levels of five genes in COAD-Colon Adenocarcinoma and READ-Rectum Adenocarcinoma database for tumor group (red) and normal group (grey). MMP9 and MET were over-expressed in tumor groups (COAD and READ), while PTGS2 (READ) and PDGFRA was under-expressed (COAD and READ) in tumor groups.

To compare the survival rates between the groups with high and low expression, Kaplan-Meier survival curves were plotted (Figure 7) and a log-rank test applied. PTGS2, MAPK1, and MET were among the 19 hub genes with a *p* value less than 0.05, which indicates that these genes may have a role in colorectal cancer survival. According to the correlation analysis (Figure 8), positive correlation exists between MMP9 & PTGS2, MMP9 & MAPK1, MMP9 & MET, PTGS2 & PDGFRA and, MET & MAPK1, whereas PGDFRA and MET have a negative correlation and there is no correlation between other gene combinations. Under expression of PTGS2 (READ) and PDGFRA (COAD and READ), over expression of MET & MMP9 (COAD and READ) were observed in the expression analysis using boxplots (Figure 9) of genes between normal and tumor samples.

## Discussion

The incidence of colon cancer is increasing, along with its mortality rates (Dong et al., 2014). The cellular mechanisms involved in colon cancer constantly change during its occurrence and progression. Malignant cancer metastasizes and invades in a complicated and polytropic manner, governed by several genes (Zhang et al., 2017). To improve the survival rate of CRC patients and prevent the disease, it is imperative to understand the etiology and mechanisms of CRC progression (Dai et al., 2020). The purpose of this study was to provide insight into the mechanism responsible for the anticancer properties of andrographolide in CRC by using network pharmacology which involves investigating the links between the drug, targets, and its pathway. DEGs from datasets GSE32323 and GSE8671 were obtained using the *p* value (<0.01) and Log_2_FC (≥2) cut off. The DEGs with positive Log_2_FC values were considered upregulated and negative Log_2_FC values as downregulated genes. The integrated target genes of CRC downloaded from both disease database and expression datasets (DEGs from datasets GSE32323 and GSE8671) were intersected with the targets of andrographolide to find the common genes shared by CRC and andrographolide. 63 genes were found to share targets with both andrographolide and CRC. 19 hub genes were identified from the PPI network with the help of degree cutoff and module score. According to GO functional enrichment analysis the hub genes were associated with GO terms such as regulation of cell migration, ERK1 and ERK2 cascade, smooth cell proliferation, apoptosis, macromolecular complex, receptor complex, membrane raft, cytoplasm, transmembrane receptor protein tyrosine kinase activity, tyrosine kinase activity, and protein kinase activity. While the KEGG pathways associated with the hub genes include EGFR tyrosine kinase inhibitor resistance, Proteoglycans in cancer, Pathways in cancer, endocrine resistance, prostate cancer, and PI3K-Akt signaling pathway. The top three genes with the lowest binding energy in molecular docking study (PDGFRA, PTGS2, and MMP9) and three hub genes (PDGFRA, MET, and MAPK1) were found to be involved in the overall survival of colorectal patients after survival analysis. These five genes (PDGFRA, PTGS2, MMP9, MAPK1, and MET) have been recognized as the target of andrographolide that has an association with CRC, thus making them the most reliable genes that can be used in clinical settings. These five genes are considered as target genes that are targeted in CRC by andrographolide. The role of these target genes in CRC is explained below.

PDGFRA is a transmembrane protein that works as a normal receptor tyrosine kinase. It has an external ligand-binding domain and an intracellular tyrosine kinase domain. When PDGF binds to PDGFRAs, it dimerizes the receptor, activating its kinase activity. The phosphorylation of tyrosine receptor leads to cellular proliferation, survival, migration, and differentiation which is initiated by the Intracellular signaling cascades, such as Ras-MAPK (mitogen-activated protein kinase), phosphatidylinositol 3-kinase (PI3K), and phospholipase C (Qian et al., 2017). In colorectal carcinogenesis, the alteration of signaling *via* the PDGFRs family plays a significant role. Most commonly, CRC is associated with overexpression of PDGFRs in tumors. Angiogenesis, invasion, and metastasis are linked to PDGFR overexpression in CRCs, in addition to the poor survival and resistance to targeted therapy (Manzat Saplacan et al., 2017).

Cyclooxygenase-(COX) or Prostaglandin endoperoxide synthases (PTGS2) plays a significant role as a rate limiting enzyme in the conversion of arachidonic acid to prostaglandins. COX exists in two isoforms (COX-1/PTGS1 & COX-2/PTGS2) (Kunzmann et al., 2013). Even though both proteins have the same cyclooxygenase and peroxidase activity, they differ in substrates, cell expression, inhibition, intracellular location, and activation. COX-2 is not generally found in normal tissues, but it is activated by a variety of hormones, cytokines, tumor promoters, and growth factors (Hidalgo-Estévez et al., 2020). PGE2 is a major PG product of COX-2 that may regulate angiogenesis, immunity, and tumorigenesis. COX-2 may be uncontrollably elevated in carcinogenesis, either transcriptionally or post-transcriptionally 55, 56. As a result, increased COX-2 expression is a tumor diagnosis marker that is linked to patient survival. According to growing evidence COX-2 signaling appears to play an important role in the metastasis of colorectal cancer. PGE2 promotes colorectal cancer cell invasion via PI3K 71 and EGFR transactivation by SRC. Besides that, COX-2 overexpression can affect intestinal cell adhesion and promote MMP (matrix metalloproteinase) activity and therefore cancer invasion. COX-2 inhibition can prevent colorectal metastasis in humans (Sinicrope and Gill, 2004).

Matrix metalloproteinases (MMPs) regulate the degradation of extracellular matrix (ECM), which is an important stage of tumor metastasis (Duffy et al., 2000; Stetler-Stevenson and Yu, 2001). The selective proteolytic degradation of Extracellular matrix (ECM) is a critical step in tumor cell migration and invasion (Yang et al., 2014). Mechanisms including transcription, activation, and inhibition control the regulation of MMPs and over 25 types of MMPs have been discovered (Andreas Jonsson, 2018). MMP9 has been detected in substantially higher ratios in the serum of CRC patients compared to normal controls. Overexpression of the p38 gamma MAPK has been demonstrated to enhance cell invasion by increasing MMP9 transcription (Loesch et al., 2010). MMP-9 levels have been proposed as a biological predictor of prognosis in CRC and other cancers such as breast and cervical cancer (Andreas Jonsson, 2018).

MAPK1/ERK1 belongs to the Ser/Thr kinase family, which activates numerous rounds of phosphorylation-activating kinase rings from the cell surface to the nucleus (Koveitypour et al., 2019). Many biological activities, including cell proliferation, differentiation, migration, and death, are regulated by mitogen-activated protein kinase (MAPK) pathways. However, genome-wide association research undertaken in Germany found the MAPK-signaling pathways as one of the most significantly related gene markers with colorectal cancer (CRC) (Slattery et al., 2012). Hepatocyte Growth Factor/Scatter Factor (HGF/SF) belongs to mesenchymal cytokine with pleiotropic effects, including mitogenic, monogenic, and morphogenic features. c-MET (or MET) encodes a receptor tyrosine kinase for HGF/SF (Gayyed et al., 2015). Under normal physiological conditions, the interaction of HGF/cMet powerfully mediates cell division and cellular organization, such as epithelial cell proliferation and migration, angiogenesis, and wound healing. In different types of malignant tumors HGF/cMet pathway plays a key role in tumour proliferation, invasion, and metastasis (Yao et al., 2019). In colorectal cancer, c-Met is associated with tumour invasiveness and aggressiveness, as well as tumor progression and poor prognosis (Liu et al., 2015).

Among the five critical genes previously obtained from survival and molecular docking analysis, MMP9 & MET were the upregulated in tumor samples (COAD and READ), while PDGFRA (COAD and READ) & PTGS2/COX-2 (READ) were downregulated according to the expression analysis by box plots. The overexpression of MET and under expression of PDGFRA and PTGS2/COX-2 was associated with overall survival of CRC patients. The above results were supported by the correlation analysis between genes which infers that there is a positive correlation between MET and MMP9 (both the genes were overexpressed in CRC), and negative correlation between PDGFRA and MET (one gene is under expressed while the other gene is overexpressed).

## Conclusion

Overall, findings seem to indicate that andrographolide may inhibit CRC *via* a diverse set of targets and pathways. Both GO annotation and KEGG pathway enrichment suggest that andrographolide may have a therapeutic role in CRC through inhibition of cellular growth, proliferation, and migration and induction of apoptosis by targeting the hub genes (PDGFRA, PTGS2, MMP9, MAPK1, and MET) which is involved in cancer migration and invasion. Based on this multidisciplinary strategy, the current study provided a promising approach for the treatment of disease using colorectal cancer. However, to clarify the relationship between andrographolide and hub genes, along with the specific mechanisms underlying those actions, more pre and clinical experiments are necessary.

## Data Availability

The datasets presented in this study can be found in online repositories. The names of the repository/repositories and accession number(s) can be found in the article/supplementary material.

## Abbreviations

COX, colorectal cancer; CRC, cyclooxygenase; DEGs, differentially expressed genes; FDR, false discovery rate; GEO, gene expression omnibus; GO, gene ontology; KEGG, kyoto encyclopedia of genes and genomics; MMP, matrix metalloproteinase; PPI, protein-protein interaction
